# Voltammetry and Spectroelectrochemistry of TCNQ in Acetonitrile/RTIL Mixtures

**DOI:** 10.3390/molecules25020303

**Published:** 2020-01-12

**Authors:** Abderrahman Atifi, Michael D. Ryan

**Affiliations:** Chemistry Department, Marquette University, P.O. Box 1881, Milwaukee, WI 53092, USA; aatifi@udel.edu

**Keywords:** TCNQ, voltammetry, spectroelectrochemistry, ion pairs, RTIL nanodomains

## Abstract

Understanding the solvation and ion-pairing interactions of anionic substrates in room-temperature ionic liquids (RTIL) is key for the electrochemical applications of these new classes of solvents. In this work, cyclic voltammetry and visible and infrared spectroelectrochemistry of tetracyanoquinodimethane (TCNQ) was examined in molecular (acetonitrile) and RTIL solvents, as well as mixtures of these solvents. The overall results were consistent with the formation of RTIL/acetonitrile nanodomains. The voltammetry indicated that the first electrogenerated product, TCNQ^−^, was not incorporated into the RTIL nanodomain, while the second electrogenerated product, TCNQ^2−^, was strongly attracted to the RTIL nanodomain. The visible spectroelectrochemistry was also consistent with these observations. Infrared spectroelectrochemistry showed no discrete ion pairing between the cation and TCNQ^−^ in either the acetonitrile or RTIL solutions. Discrete ion pairing was, however, observed in the acetonitrile domain between the tetrabutylammonium ion and TCNQ^2−^. On the other hand, no discrete ion pairing was observed in BMImPF_6_ or BMImBF_4_ solutions with TCNQ^2−^. In BMImNTf_2_, however, discrete ion pairs were formed with BMIm^+^ and TCNQ^2−^. Density function theory (DFT) calculations showed that the cations paired above and below the aromatic ring. The results of this work support the understanding of the redox chemistry in RTIL solutions.

## 1. Introduction

The use of room-temperature ionic liquids (RTILs) has attracted considerable interest among electrochemists because of their electrical conductivity and their ability to solvate ionic species, especially anions. The effect of this solvation is seen most dramatically in the collapse of the two one-electron waves of dinitrobenzene into a single two-electron wave in RTILs [1,2]. In general, anionic reduction products are better solvated by the cation of the RTIL than their neutral precursors. While the collapse of two one-electron waves into a single two-electron wave is an extreme example, the potential shifts are significant for a variety of organic compounds. Nikitina et al. [3] examined the variation in redox potentials for redox systems that undergo sequential single-electron transfers. Generally, the difference between the waves (∆E_12_° = E_1_° − E_2_°, where 1 and 2 are sequential redox couples) was smaller for RTILs as compared to molecular solvents, due to the increased solvation of the more negatively charged species. The variation in the ∆E_12_° values as a function of %RTIL has been studied for several substrates by Atifi and Ryan [2,4,5,6,7] and others [8].

Szwarc [9] defined two types of ion pairs: contact or loose ion pairs. Contact ion pairs (discrete ion pairs) favor a defined geometric arrangement between the anion and cation. For loose ion pairs (general solvation), the presence of solvent between the ions prevents such direct atomic interactions, and the arrangement of cations around the anions is best described as an ionic cloud around the anion. The formation of tight ion pairs has the greatest effect on the spectroscopic properties of the substrate [9], especially the vibrational spectra. For loose ion pairs, a continuum model best describes the interactions. From voltammetric data, one cannot determine whether tight or loose ion pairs are formed. In pure RTILs, the voltammetric behavior may be due to general solvation of the substrate by the RTIL (loose ion pair), or it might be due to the formation of discrete and relatively long-lasting ion pairs (at least long enough to be observed spectroscopically) with a specific geometric structure and atomic interactions (i.e., tight ion pairs).

**Figure 1:**
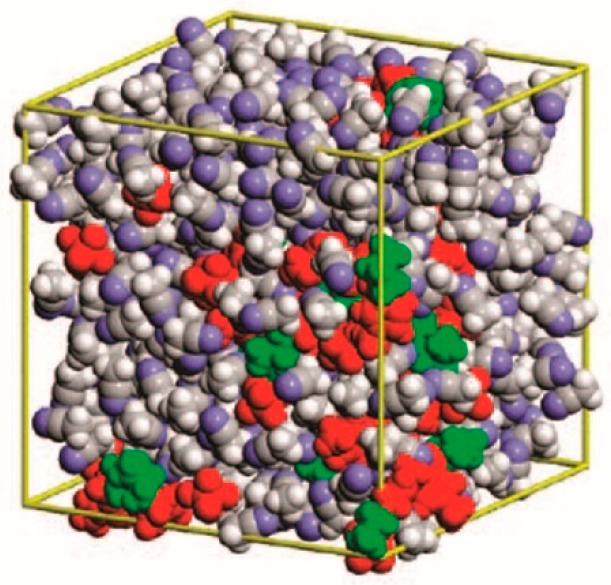
Mixture of acetonitrile with 20% (v/v) BMImPF_6_. Red: BMIm^+^, Green: PF_6_^−^, Blue/white: acetonitrile. Adapted with permission from *Acc. Chem. Res.* (2007) 40, 1087–1096. Copyright 2007 American Chemical Society.

Recent work has shown that mixed molecular/RTIL solutions are often not homogeneous solutions, but can lead to the formation of aggregates (nanodomains) [10,11,12,13]. Figure 1 shows the formation of RTIL nanodomains in a molecular-solvent-rich solution. The substrate can partition between these two nanodomains. Within each nanodomain, the solute will experience different solvation interactions and reactivity [7]. There are also structural heterogeneities within the RTIL itself, where there are more polar regions containing the anion and less polar regions where the alkyl side chains reside [13]. We might expect the electrogenerated anionic species to be within the more polar region, but they might not be able to displace the strong interactions between the cation and the RTIL anion. As a result, the electrogenerated anion might not be able to form a discrete ion pair with the RTIL cation. For RTIL-rich solutions, the solvents will reverse themselves with molecular solvent islands in a sea of RTIL (Supplementary Figure S1 for RTIL-rich solutions). Since the nanodomains are small, the RTIL and molecular solvent nanodomains will be at equilibrium. The molecular properties of the substrates can affect the way the aggregates interact with the redox products. The ability of the substrate to penetrate and interact with the RTIL domain will affect the observed potential shifts. For RTILs with planar cations (e.g., BMIm^+^), planar substrates will interact more strongly than spherical substrates [4,5]. For RTILs with more flexible cations (e.g., tetraalkylammonium ions), spherical substrates can interact more strongly [5]. Generally, the second reduction wave shifts more than the first wave, but the opposite effect can be observed for Ni(OEPone) (OEPone = octaethylporphinone) reduction [4]. In this case, a strong interaction between the carbonyl group of the Ni(OEPone)^−^ anion and the RTIL leads to the formation of two ν_CO_ bands, one for Ni(OEPone)^−^ in the THF domain, and a second band for Ni(OEPone)^−^ in the RTIL domain. This interaction leads to a considerable stabilization of the anion.

**Scheme 1:**
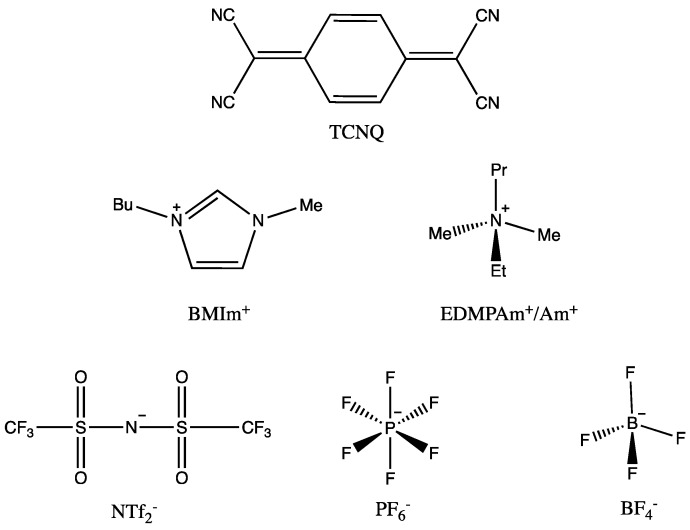
Structures of TCNQ and the room-temperature ionic liquids (RTILs) used in this work. TCNQ = tetracyanoquinodimethane; BMIm^+^: 1-butyl-3-methyl imidazolium; EDMPAm^+^: ethyldimethylpropyl ammonium; NTf_2_^−^: bis(trifluoromethylsulfonyl)imide.

In order to probe the effect of substrate structure on its behavior in mixed acetonitrile/RTIL mixture, voltammetric and spectroelectrochemical study of TCNQ was carried out herein. Scheme 1 shows the molecular structure of TCNQ and the anions/cations of the RTILs used in this work.

TCNQ is reduced in two sequential one-electron transfers to form a radical anion and a dianion [14,15,16].

(1) $$\mathrm{TCNQ}\;+\;e^{-}\;\to \;{\mathrm{TCNQ}}^{-}$$

(2) $${\mathrm{TCNQ}}^{-}\;+\;e^{-}\;\to \;{\mathrm{TCNQ}}^{2-}$$

The resonance Raman spectroscopy of electrogenerated TCNQ^−^ was reported by Jeanmaire and van Duyne [14]. Suchanski and van Duyne [17] were also able to generate the dianion, which was colorless, preventing them from obtaining a resonance Raman spectrum at that time. The electron transfer rate for the first wave was obtained by Sharp [18]. The infrared spectroelectrochemistry of TCNQ in acetonitrile and DMSO was studied by Khoo et al. [19]. Flower and Mamantov [20] and Bellec et al. [21] also studied the FTIR spectroelectrochemistry of TCNQ.

The FTIR spectroelectrochemistry of TCNQ and its reduction products had 1–2 bands between 2000–2300 cm^−1^. TCNQ had one band at 2224 cm^−1^ for two degenerate states (ν_19_ and ν_33_). Upon reduction, the two levels split and gave separate bands. For TCNQ^−^, the bands were observed at 2156 and 2182 cm^−1^ in acetonitrile/tetrabutylammonium perchlorate (TBAP). Further reduction to TCNQ^2−^ caused an additional downshift to 2106 and 2153 cm^−1^. Addition of Na^+^, Li^+^, and TEA^+^ ions caused no significant changes in the TCNQ^−^ spectrum. For the dianion, though, the bands were upshifted in the presence of Na^+^ and Li^+^ due to ion pairing. A larger shift was observed for the lithium salt, with the 2153 cm^−1^ band upshifting to 2166 cm^−1^, and the 2106 cm^−1^ band upshifting to 2118 cm^−1^. No shifts in the dianion spectra were observed when acetonitrile was replaced with DMSO. The observed shifts were interpreted on the basis of ion pairing. The LUMO for TCNQ was antibonded with respect to the CN triple bond, which led to the downshift of the ν_CN_ stretching bands upon reduction. If the cation ion paired at the nitrogen atoms, this would enhance the anti-bonding interaction, leading to a downshift in the ν_CN_, as was observed for the reduction. On the other hand, if the cation was over the center of the six-member ring, this interaction increased the contributions from the resonance structures where the CN triple bond was intact, leading to the observed upshift. Therefore, alkali ions appeared to form tight ion pairs over the center of the six-member ring of the TCNQ dianion. The other important conclusion of this work was that the splitting of the ν_CN,str_ was independent of the amount of ion pairing. Theoretical and experimental evidence has shown that the splitting is proportional to the charge per π-bonded atom in the molecule. Little work has been done on the voltammetry of TCNQ in RTILs outside of the work Zhao et al. [22].

## 2. Results and Discussion

**Figure 2:**
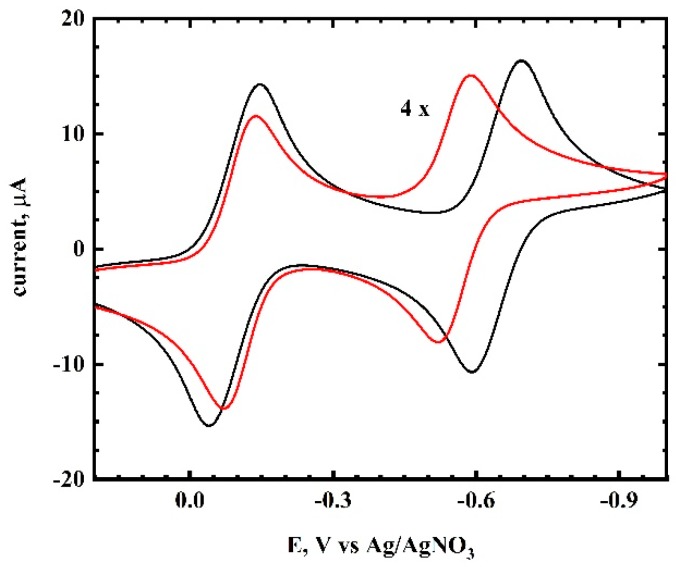
Cyclic voltammetry of TCNQ in acetonitrile (black trace) and in 20% AmNTf_2_/acetonitrile. Scan rate: 100 mV/s. Working electrode: platinum. Electrolyte: 0.10 M tetrabutylammonium perchlorate (TBAP).

Cyclic Voltammetry of TCNQ in Acetonitrite/RTIL Mixtures. The cyclic voltammetry of TCNQ in acetonitrile occurs in two one-electron steps (Figure 2). The first wave had an E°_1_ = −0.094 V and the second wave had an E°_2_ = −0.643 V, giving an ∆E°_12_ value of 550 mV. The cyclic voltammetry results of TCNQ in several RTILs are shown in Supplementary Figure S2, and the results are summarized in Table 1. The ∆E°_12_ for TCNQ decreased from 550 mV in acetonitrile to 427 mV in BMImBF_4_. The first wave was not significantly affected by the RTIL, but the second wave shifted to more positive potentials. These results show that the dianion was solvated much more strongly in the RTIL than in acetonitrile.

**Figure 3:**
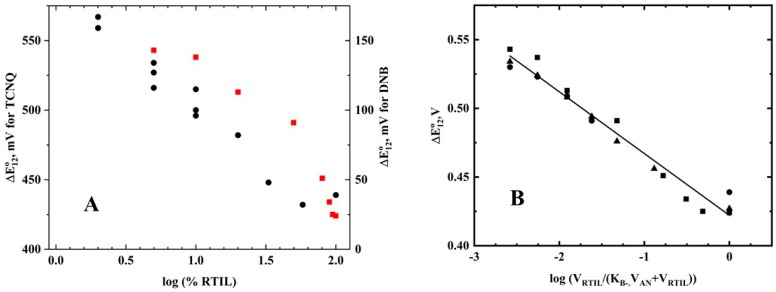
(**A**) ∆E°_12_ values for TCNQ (red) and 1,4-dinitrobenzene (black) in acetonitrile/AmNTf_2_ mixtures as a function of log (%RTIL). Dinitrobenzene data from Reference [2]. (**B**) ∆E°_12_ values for TCNQ in acetonitrile/RTIL mixtures as a function of Equation (3). (ν) AmNTf_2_; (●) BMImPF_6_; (▲) BMImBF_4_. Electrolyte in acetonitrile and mixed acetonitrile/RTIL solutions: 0.10 M TBAP.

In mixed molecular solvent/RTIL solutions, the substrate could be solvated by a combination of molecular solvent and RTIL nanodomains (Figure 1). The cyclic voltammetry of TCNQ in acetonitrile and 20% AmNTf_2_ is shown in Figure 2. As was observed for 1,4-dinitrobenzene in similar solutions, the E°_1_ was not significantly affected by the presence of the RTIL, while the second wave (E°_2_) was shifted to more positive potentials [2]. The results for mixed acetonitrile/AmNTf_2_ solutions are summarized in Table 2, and similar results were observed for BMImBF_4_ and BMImPF_6_ (Table S1). For all these mixtures, the E°_1_ values were relatively insensitive to the %RTIL. On the other hand, the E°_2_ values shifted significantly to more positive potentials as the %RTIL increased. This was qualitatively similar to the results observed for 1,4-dinitrobenzene [2]. In order to evaluate these results quantitatively, we needed to use the difference in potentials between two waves, ∆E°_12_, as the E° values themselves also depended upon the reference potential, which varied with the %RTIL. The shifts in the ∆E°_12_ values for TCNQ are shown in Table 2 and Figure 3A (red squares), along with the data for 1,4-dinitrobenzene (DNB) in acetonitrile/RTIL solutions (black circles) [2]. Unlike the DNB results, the TCNQ data showed a non-linear relationship between the log(%RTIL) and the ∆E°_12_ values.

The relationship between %RTIL and E°_2_ was derived in Reference [2], and is shown in Equation (3).

(3) $$E_{2,\mathrm{mixture}}^{{}^{\circ}}=E_{2,\mathrm{RTIL}}^{{}^{\circ}}-0.059\log\left(\frac{V_{\mathrm{RTIL}}}{K_{B-.}V_{\mathrm{AN}}+V_{\mathrm{RTIL}}}\right)$$

where K_B−_ is the partitioning coefficient between the acetonitrile (AN) and the RTIL nanodomains and is equal to [B^−^]_AN_/[B^−^]_RTIL_, and B^−^ is the first reduction product. As E°_1_ had little dependence on the %RTIL, most of the shift in the ∆E°_12_ was due to shifts in the second reduction. For the DNB data, K_B−_ was assumed to be 1, and the term in brackets in Equation (3) was reduced to the %RTIL (v/v). If K_B−_ was greater than 1 (radical anion more soluble in acetonitrile nanodomain), a non-linear relationship between E°_2_ (and hence ∆E°_12_) was observed. The data for all the RTIL data for TCNQ are plotted in Figure 3B using Equation (3) and K_B−_ = 20 (K_B_^−^ should be considered a minimum value, as the equation is relatively insensitive to K_B_^−^ for larger values). The slope was found to be 45 mV—somewhat lower than the theoretical value, but reasonable given the assumptions of the derivation. The results indicate that TCNQ^−^ was found almost exclusively in the acetonitrile phase (≥95%). Because the E°_1_ was independent of %RTIL, this means that K_B_ was also large. The cyclic voltammetric data indicate that the following electron-transfer processes occurred:

(4) $${\mathrm{TCNQ}}_{\mathrm{AN}}\;+\;e^{-}\;⇋\;{\mathrm{TCNQ}}^{-}{}_{\mathrm{AN}}$$

(5) $${\mathrm{TCNQ}}^{-}{}_{\mathrm{AN}}\;+\;e^{-}\;⇋\;{\mathrm{TCNQ}}^{2-}{}_{\mathrm{RTIL}}$$

A linear relationship between log(%RTIL) and ∆E°_12_ was observed for NiOEP oxidation and Fe(FP)(Cl) reduction, while non-linearity, which was observed for TCNQ, was also seen for Fe(FP)(ClO_4_) (FP = tetrakis(pentafluorophenyl)porphyrin) [6]. Both linear and non-linear behavior was observed for the different reduction waves of C_60_ [5].

**Figure 4:**
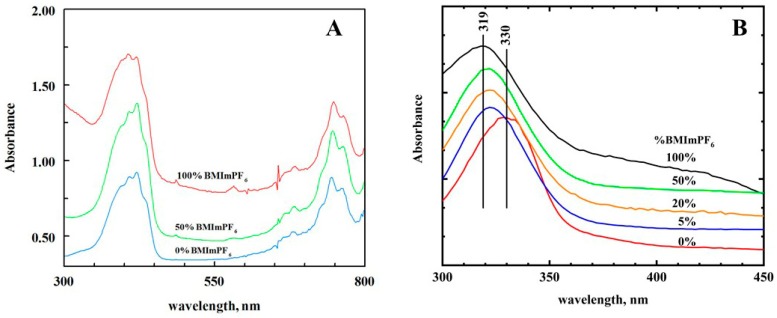
(**A**). Visible spectra of TCNQ^−^ in acetonitrile (blue), 50% acetonitrile/BMImPF_6_ (green), and BMImPF_6_ (red). (**B**). Visible spectra of TCNQ^2−^. %BMImPF_6_ in acetonitrile: 0% (red), 5% (blue), 20% (orange), 50% (green), 100% (black). Acetonitrile solutions contained 0.10 M TBAP.

Visible Spectroelectrochemistry. A UV/visible spectroelectrochemical analysis of TCNQ in acetonitrile was carried out (Supplementary Figure S3). The UV/visible spectra for TCNQ, TCNQ^−^, and TCNQ^2−^ were consistent with previous work [14,17]. The UV/visible spectra for TCNQ and TCNQ^−^ were virtually identical in acetonitrile and BMImPF_6_ (Figure 4A for TCNQ^−^). These results were consistent with the cyclic voltammetric data, which showed that TCNQ^−^ was not incorporated into the RTIL nanodomain. This would indicate weak interactions between the RTIL and TCNQ^−.^ in moving from acetonitrile to the RTIL. On the other hand, the dianion TCNQ^2−^ strongly interacted with the RTIL. Visible spectroelectrochemistry showed that the 330 nm band for TCNQ^2−^ was blue-shifted to 319 nm as the %RTIL increased (Figure 4B). The largest blue shift was seen between 0% and 5% RTIL in acetonitrile, and was consistent with the strong attraction of TCNQ^2−^ for the RTIL nanodomain. This also supports the idea that RTIL nanodomains are formed at relatively low concentrations of RTIL in acetonitrile. For AmNTf_2_, however, there was very little shift in the λ_max_ (328 nm).

**Figure 5:**
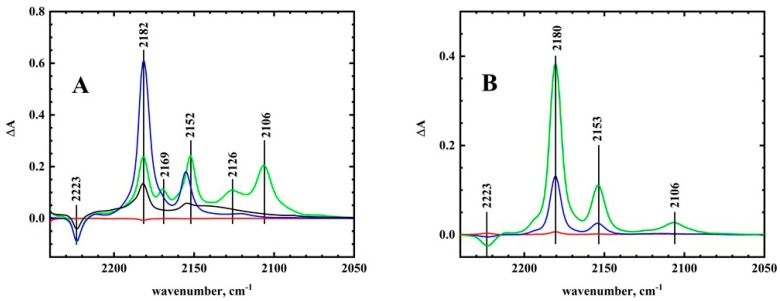
Infrared spectroelectrochemistry of TCNQ. (**A**) In acetonitrile. Initial difference spectrum (red); TCNQ^−^ (blue); TCNQ^2−^ (green); end of scan (black). Electrolyte: 0.10 M TBAP (**B**) In BMImBF_4_. Initial difference spectrum (red); TCNQ^−^ (blue); TCNQ^2−^ (green).

Infrared Spectroelectrochemistry. The FTIR spectroelectrochemistry of TCNQ were measured in acetonitrile/RTIL solutions. The different infrared spectroelectrochemical spectra of TCNQ in acetonitrile are shown in Figure 5A. The formation of TCNQ^−^ is shown in the blue spectrum with positive bands at 2182 and 2155 cm^−1^, and a negative band at 2223 cm^−1^ for the disappearance of TCNQ. This spectrum was essentially the same as previously reported spectra [19]. Further reduction to form TCNQ^2−^ can be seen in Figure 5A (green trace). The 2182 cm^−1^ band downshifted to 2152 cm^−1^, while the 2155 cm^−1^ downshifted to 2106 cm^−1^. These bands were also observed by Khoo et al. [19]. In addition to these bands, two additional bands were seen at 2169 and 2126 cm^−1^. These bands were not seen by Khoo et al. [19], and the spectra of Flowers and Mamantov [20] were ambiguous because of their low resolution. Recent infrared spectroelectrochemistry of TCNQ by Bellec et al. [21] did show those two bands in their figure (but they did not report the energies in the text). The 2169 cm^−1^ band was close to the values for the ion pair bands for Na^+^ and Li^+^ (2156 and 2166 cm^−1^) [19], and the 2126 cm^−1^ band was similar to the 2118 cm^−1^ band in the presence of Li^+^. In the work of Khoo et al., the new bands completely replaced the band for the free dianion, while with TBA^+^, both bands were observed, indicating a weaker interaction for the tetraalkylammonium ion. We repeated the experiment in THF, and the same spectra were obtained (Supplementary Figure S4). In our experiment, tight ion pairs between TBA^+^ and TCNQ^2−^ were observed, but were not the majority species in solution.

The structure of the ion pair between TBA^+^ and TCNQ^2−^ was studied using density functional theory (DFT) calculations. The result is shown in Supplementary Figure S5. The best structure showed that the cations were situated above and below the ring of the TCNQ^2−^ ion, as predicted by Khoo et al. for alkali ions [19]. For the tetraalkylammonium ions, the calculations of Fry [23] showed that the positive charge is carried by the α-methylene protons. Longer alkyl chains had little effect on the structure. Fry [24] also showed that the tetraalkylammonium ion also ion pairs above the aromatic ring. Both results were consistent with our calculated structure.

The infrared spectroelectrochemistry experiment was then repeated in neat BMImBF_4_, and the results are shown in Figure 5B. During the electrolysis, the two bands for TCNQ^−^ (2153 and 2180 cm^−1^, Figure 5B—blue trace) appeared first. Further reduction led to the formation of the 2106 cm^−1^ TCNQ^2−^ band. Because of the high resistance of the RTIL, complete reduction to TCNQ^2−^ did not occur. Unlike the electrolysis in acetonitrile, the bands for tight ion pairs, especially the 2126 cm^−1^ band, were not observed. The spectral changes were reversible and the radical anion and neutral spectra were obtained upon reversal of the scan. The absence of discrete ion pairs in RTILs can be understood by examining the structure of the RTILs themselves. Unlike molecular solvents, RTILs provide solvation and charge neutralization at the same time. In RTILs, ion pairs are highly transient. Discrete stable structures are needed in order to observe the ion pair spectroscopically. In acetonitrile, the electrolyte is mostly dissociated, and the TCNQ^2−^ anion does not have to compete with the electrolyte counterion to form an ion pair. In RTILs, this is not true, and the anionic substrate must displace the RTIL counterion. For small, basic, and symmetrical anions such as PF_6_^−^ and BF_4_^−^, the RTIL itself forms a tight structure [25]. This attraction then forces TCNQ^2−^ into the less polar region of the RTIL [13]. In fact, this was most apparent for the radical anion, which was more favorably solvated in the acetonitrile phase than in the RTIL phase.

**Figure 6:**
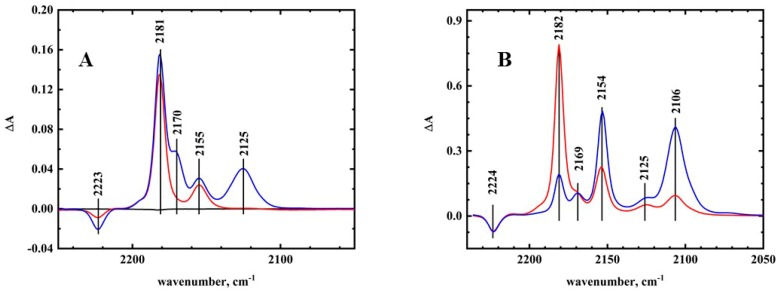
Infrared spectroelectrochemistry of TCNQ. (**A**) In BMImNTf_2_. Initial difference spectrum (black); TCNQ^−^ (red); TCNQ^2−^ (blue). (**B**) In 50% BMImPF_6_/acetonitrile. TCNQ^−^ (with some TCNQ^2−^) (red); TCNQ^2−^ (blue). Electrolyte: 0.10 M TBAP.

The balance between discrete ion pairs versus general solvation can be seen in the infrared spectroelectrochemistry of another RTIL, BMImNTf_2_. These results are shown in Figure 6A. The larger and less symmetrical ion NTf_2_^−^ is known to form a looser ion pair structure in the RTIL than BF_4_^−^/PF_6_^−^. The spectrum for the first reduction was essentially the same as was seen for acetonitrile and BMImPF_6_, with new bands for the radical anion appearing at 2181 and 2155 cm^−1^. The second reduction, however, showed significant changes. The bands for the dianion were observed at 2170 and 2125 cm^−1^, with no band observed at 2106 cm^−1^. This upshift of the dianion bands was consistent with ion pairing between the solvent cation and TCNQ^2−^, as was observed for TBA^+^ in acetonitrile. Unlike BMImPF_6_/BMImBF_4_, TCNQ^2−^ was able to enter the more polar region of the RTIL, displacing NTf_2_^−^ [13]. Reversal of the potential to the initial potential showed that the reduction was reversible, with the 2170 and 2125 cm^−1^ bands disappearing upon oxidation.

The spectroelectrochemistry of TCNQ was also examined in acetonitrile/50% BMImPF_6_. The results are shown in Figure 6B. The main features in the spectrum were similar to acetonitrile, including the presence of the ion pairing bands at 2125 and 2169 cm^−1^. These bands were weaker, however, than in Figure 5A, indicating that they were due to residual TCNQ^2−^ in the acetonitrile phase. This was expected, as most of the TCNQ^2−^ species were incorporated into the RTIL nanodomain. The free TCNQ^2−^ had the same spectrum in both acetonitrile and RTIL nanodomain. Since the TCNQ^2−^ bands were almost identical in acetonitrile and BMImPF_6_, this would indicate that TCNQ^2−^ in the RTIL nanodomains of the acetonitrile/RTIL mixture had the same solvation as in the pure RTIL solvent.

The results of this electrochemical/spectroscopic study showed that the incorporation of the electrogenerated anions into RTIL nanodomains or solvent is not a simple matter. The obvious expectation was that the anionic substrate would replace the RTIL counterion in the RTIL domain. This may have occurred in some instances, but not in all. As discussed earlier, RTILs are not homogeneous random mixtures of anions and cations, but contain a defined structure over a certain distance. As a result, within the pure RTIL, there are ionic regions where the anions and cations are in close proximity, and other regions which are reasonably non-polar, caused by the aggregation of the alkyl groups. Small, spherical, and basic anions such as BF_4_^−^/PF_6_^−^ can prevent the electrogenerated anion from forming discrete ion pairs with the RTIL cation. Therefore, if the interaction between TCNQ^2−^ and the RTIL cation is nonspecific and there is a tight ion pair between the RTIL anion and cation, the TCNQ^2−^ will find regions of minimum energy away from the cation. In that case, we would see no significant changes in the vibrational spectrum. This was the case with BMImPF_6_, where tight ion pairing between BMIm^+^ and PF_6_^−^ prevented a tight ion pair with TCNQ^2−^, despite being doubly charged. Because of the delocalization of the charge in TCNQ^2−^, the effect of the dianion was probably minimized. On the other hand, in BMImNTf_2_, the weaker ion pairing between BMIm^+^ and NTf_2_^−^ allowed the TCNQ^2−^ species to form a tight ion pair with BMIm^+^, leading to significant changes in the infrared spectrum. The second important conclusion of this work is confirmation of the formation of RTIL nanodomains at relatively low concentrations of RTIL (5%), based on visible spectra.

In assessing the formation of ion pairs, it is important to distinguish between discrete ion pairs and general ionic solvation. Both these forms of ion pairing would yield similar voltammetric behavior of the redox potential. In this case, even when discrete ion pairing was observed, these ion pairs were not the majority of the species present for the dianion, except for BMImNTf_2_. We could not use electrochemical data (shift of the E° value) alone to identify ion pairs. Identification of discrete ion pairs required examination of spectroscopic signatures to ascertain whether discrete ion pairs were present.

## 3. Experimental Section

Chemicals. High purity RTILs 1-butyl-3-methylimidazolium hexafluorophosphate (BMImPF_6_), 1-butyl-3-methylimidazolium tetrafluoroborate (BMImBF_4_), and 1-butyl-3-methylimidazolium bis(trifluoromethylsulfonyl)-amide (BMImNTf_2_) were purchased from Merck and TCI and used without further purification, except as noted in the text. Ethyldimethylpropylammonium bis(trifluoromethylsulfonyl)imide (AmNTF_2_), tetrabutylammonium perchlorate (TBAP), 7,7,8,8-tetracyanoquinodimethane (TCNQ), and anhydrous acetonitrile (ACN, 99.8%), were purchased from Sigma-Aldrich Chemical Co., St. Louis, MO, USA Anhydrous tetrahydrofuran (THF) was refluxed in the presence of sodium and benzophenone under nitrogen until the solution was blue. Other molecular solvents were used as received.

Instrumentation. Cyclic voltammetry was carried out on a platinum electrode (1.6 mm or 10 μ), and a platinum wire was used as auxiliary electrode. Potentials were measured relative to the Ag/AgNO_3_ (in CH_3_CN) reference electrode. The voltammetric measurements were carried out using a Model 600D Series Electrochemical Analyzer/Workstation (CHI Version 12.06, CH Instruments, Austin, TX, USA).

A low-volume, thin-layer quartz cell, which was purchased from Bioanalytical Systems (West Lafayette, IN, USA), was used for UV/visible spectroelectrochemical experiments. A platinum mesh was used as the working electrode, a platinum wire was used as an auxiliary electrode, and the reference electrode was an Ag/AgNO_3_ (in CH_3_CN) electrode. UV/visible spectra were recorded on a HP8452A diode array spectrophotometer (Agilent Technologies, Santa Clara, CA, USA). A homemade infrared spectroelectrochemical cell was made by modifying a Wilmad semi-permanent cell. The Teflon spacer between two CaF_2_ windows was replaced by a polyethylene spacer into which the working, reference, and auxiliary electrodes were melt-sealed. The working and auxiliary electrodes were fabricated from 100 mesh platinum gauze (Sigma-Aldrich Chemical Co.), and a silver wire (0.05 mm diameter, Johnson Matthey, London, UK) was used as a pseudo reference electrode. The infrared spectra were obtained using 64 scans at 2 cm^−1^ resolution, recorded with a Thermo Nicolet-FTIR spectrophotometer (Model 670 Nexus, Thermo Fisher Scientific, Waltham, MA, USA) with a MCT detector.

Procedures. For electrochemical experiments, all solutions were prepared and filled into cells in the glovebox under an argon environment. Tetrabutylammonium perchlorate was used as electrolyte in acetonitrile experiments. UV/visible and FTIR spectroelectrochemical experiments were carried out by either scanning or stepping the potentials at the corresponding waves. For UV/visible experiments, the entrance window of the cell was masked so that the spectral beam passed only through the working electrode.

For mixtures of acetonitrile/RTIL solvents, a total volume of 0.3–0.5 mL was used for voltammetry, and 0.1–0.2 mL for spectroelectrochemistry. Water was removed from the RTIL by passing N_2_ over the solvent, which was heated at 70 to 90 °C. The residual water in the RTIL was evaluated by monitoring the stripping peak on a gold electrode due to water [26].

Computational methods. Electronic structure and vibrational spectral calculations were carried out using the m06 functionals and the TZVP basis set, except as noted in the text, using the Gaussian 16 suite of programs [27]. All calculations converged using the tight optimization criteria. Solvation effects were calculated in Gaussian 16 using the PCM [28].

## Figures and Tables

**Table 1:** Cyclic voltammetry of TCNQ in acetonitrile and several RTILs. Scan rate = 100 mV/s; working electrode was platinum. Electrolyte in acetonitrile: 0.10 M TBAP.

Solvent	E°_1_, V vs. Ag/AgNO_3_	E°_2_, V vs. Ag/AgNO_3_	∆E°_12_, mV
Acetonitrile	−0.094	−0.644	550
BMImNTf_2_	−0.100	−0.550	450
AmNTf_2_	−0.106	−0.540	434
BMImBF_4_	−0.096	−0.523	427
BMImPF_6_	−0.121	−0.562	441

**Table 2:** Cyclic voltammetry at 100 mV/s for TCNQ in acetonitrile/AmNTf_2_.

%AmNTf_2_	E°_1_, V vs. Ag/AgNO_3_	E°_2_, V vs. Ag/AgNO_3_	∆E°_12_, mV
0	−0.094	−0.643	549
5	−0.087	−0.630	543
10	−0.082	−0.620	538
20	−0.090	−0.603	513
50	−0.087	−0.578	491
80	−0.103	−0.554	451
90	−0.112	−0.546	434
95	−0.111	−0.536	425
100	−0.112	−0.536	424

## Supplementary Materials

The following are available online, Table S1. Cyclic voltammetry of TCNQ in mixtures of acetonitrile and BMImPF_6_ or BMImBF_4_, Figure S1. Mixture of acetonitrile with 60% (v/v) BMImPF_6_, Figure S2. Cyclic voltammetry of TCNQ in AmNTf_2_, BMImBF_4_, BMImPF_6_, BMImNTf_2_ and Acetonitrile, Figure S3. Visible spectroelectrochemistry of TCNQ in acetonitrile, Figure S4. Infrared spectroelectrochemistry of TCNQ in THF, Figure S5. DFT structure of the tetramethylammonium-TCNQ^2−^ ion pair.

## References

[1] Fry A.J. (2003). Strong ion-pairing effects in a room temperature ionic liquid. J. Electroanal. Chem..

[2] Atifi A., Ryan M.D. (2014). Electrochemistry and Spectroelectrochemistry of 1,4-Dinitrobenzene in Acetonitrile and Room Temperature Ionic Liquids: Ion Pairing Effects in Mixed Solvents. Anal. Chem..

[3] Nikitina V.A., Gruber F., Jansen M., Tsirlina G.A. (2013). Subsequent redox transitions as a tool to understand solvation in ionic liquids. Electrochim. Acta.

[4] Atifi A., Ryan M.D. (2015). Spectroscopic Evidence of Nanodomains in THF/RTIL Mixtures: Spectroelectrochemical and Voltammetric Study of Nickel Porphyrins. Anal. Chem..

[5] Atifi A., Ryan M.D. (2016). Influence of RTIL Nanodomains on the Voltammetry and Spectroelectrochemistry of Fullerene C_60_ in Benzonitrile/Room Temperature Ionic Liquids Mixtures. Electrochim. Acta.

[6] Atifi A., Ryan M.D. (2017). Altering the Coordination of Iron Porphyrins by Ionic Liquid Nanodomains in Mixed Solvent Systems. Chem. Eur. J..

[7] Atifi A., Mak P.J., Ryan M.D. (2019). Proton-coupled reduction of an iron nitrosyl porphyrin in the protic ionic liquid nanodomain. Electrochim. Acta.

[8] Xie Y., Li D., Jin B. (2016). In situ FT-IR spectroelectrochemical study of the reduction of 1,4-dinitrobenzene in room-temperature ionic liquids. J. Electroanal. Chem..

[9] Szwarc M. (1969). Ions and ion pairs. Acc. Chem. Res..

[10] Li W., Zhang Z., Zhang J., Han B., Wang B., Hou M., Xie Y. (2006). Micropolarity and aggregation behavior in ionic liquid + organic solvent solutions. Fluid Phase Equilib..

[11] Shin D.N., Wijnen J.W., Engberts J.B.F.N., Wakisaka A. (2001). On the Origin of Microheterogeneity: A Mass Spectrometric Study of Dimethyl Sulfoxide-Water Binary Mixture. J. Phys. Chem. B.

[12] Bhat M.A. (2012). Mechanistic, kinetic and electroanalytical aspects of quinone-hydroquinone redox system in N-alkylimidazolium based room temperature ionic liquids. Electrochim. Acta.

[13] Pádua A.A.H., Costa Gomes M.F., Canongia Lopes J.N.A. (2007). Molecular Solutes in Ionic Liquids: A Structural Perspective. Acc. Chem. Res..

[14] Jeanmaire D.L., Van Duyne R.P. (1976). Resonance Raman spectroelectrochemistry. 2. Scattering spectroscopy accompanying excitation of the lowest ^2^B_1u_ excited state of the tetracyanoquinodimethane anion radical. J. Am. Chem. Soc..

[15] Peover M.E. (1964). Polarographic study of some aromatic molecular complexes. Trans. Faraday Soc..

[16] Acker D.S., Hertler W.R. (1962). Substituted quinodimethans. I. Preparation and chemistry of 7,7,8,8-tetracyanoquinodimethan. J. Am. Chem. Soc..

[17] Suchanski M.R., Van Duyne R.P. (1976). Resonance Raman spectroelectrochemistry. IV. The oxygen decay chemistry of the tetracyanoquinodimethane dianion. J. Am. Chem. Soc..

[18] Sharp M. (1978). The determination of electron transfer rates for several quinonoid compounds at platinum and gold electrodes. J. Electroanal. Chem..

[19] Khoo S.B., Foley J.T., Korzeniewski C., Pons S. (1987). An infrared spectroelectrochemical investigation of the ion pairing reactions of the anions and dianions of TCNE and TCNQ. J. Electroanal. Chem..

[20] Flowers P.A., Mamantov G. (1989). Thin-layer transmittance cell for infrared spectroelectrochemistry. Anal. Chem..

[21] Bellec V., De Backer M.G., Levillain E., Sauvage F.X., Sombret B., Wartelle C. (2001). In situ time-resolved FTIR spectroelectrochemistry: Study of the reduction of TCNQ. Electrochem. Commun..

[22] Zhao C., Bond A.M., Compton R.G., O‘Mahony A.M., Rogers E.I. (2010). Modification and Implications of Changes in Electrochemical Responses Encountered When Undertaking Deoxygenation in Ionic Liquids. Anal. Chem..

[23] Fry A.J. (2013). Computational Studies of Ion Pairing. 7. Ion-Pairing and Association Effects between Tetraalkylammonium Ions and Nitrobenzene Redox Species. Ion Pairing to Neutral Substances. J. Org. Chem..

[24] Fry A.J. (2010). The effect of tetramethylammonium ion on the voltammetric behavior of polycyclic aromatic hydrocarbons: Computations explain a long-standing anomaly. Phys. Chem. Chem. Phys..

[25] Hapiot P., Lagrost C. (2008). Electrochemical Reactivity in Room-Temperature Ionic Liquids. Chem. Rev..

[26] Zhao C., Bond A.M., Lu X. (2012). Determination of Water in Room Temperature Ionic Liquids by Cathodic Stripping Voltammetry at a Gold Electrode. Anal. Chem..

[27] Frisch M.J., Trucks G.W., Schlegel H.B., Scuseria G.E., Robb M.A., Cheeseman J.R., Scalmani G., Barone V., Petersson G.A., Nakatsuji H. (2016). Gaussian 16 Rev. B.01.

[28] Tomasi J., Persico M. (1994). Molecular Interactions in Solution: An Overview of Methods Based on Continuous Distributions of the Solvent. Chem. Rev..

