# Supplementary material for: Voltammetry and Spectroelectrochemistry of TCNQ in Acetonitrile/RTIL Mixtures
Source: Molecules. 2020 Jan 12;25(2):303. doi: 10.3390/molecules25020303 (PMC7024151; doi:10.3390/molecules25020303)

Voltammetry and Spectroelectrochemistry of TCNQ in Acetonitrile/RTIL Mixtures

Abderrahman Atifi and Michael D. Ryan*

Marquette University, Chemistry Department, PO Box 1881, Milwaukee, WI 53092 USA
E-mail: Michael.ryan@marquette.edu

Table of Contents.

Table S1. Cyclic voltammetry of TCNQ in mixtures of acetonitrile and $BMImPF_6$ or $BMImBF_4$ 2

Figure S1. Mixture of acetonitrile with 60% (v/v) $BMImPF_6$ 3

Figure S2. Cyclic voltammetry of TCNQ in $AmNTf_2$, $BMImBF_4$, $BMImPF_6$, $BMImNTf_2$ and Acetonitrile 4

Figure S3. Visible spectroelectrochemistry of TCNQ in acetonitrile 5

Figure S4. Infrared spectroelectrochemistry of TCNQ in THF 6

Figure S5. DFT structure of the tetramethylammonium-$TCNQ^{2-}$ ion pair 7

Table S1. Cyclic voltammetry of TCNQ in mixtures of acetonitrile and $BMImPF_6$ or $BMImBF_4$. Acetonitrile solutions contained 0.10 M TBAP

Cyclic voltammetry of TCNQ in acetonitrile/$BMImPF_6$

%$BMImPF_6$	$E°_1$, V vs Ag/$AgNO_3$	$E°_2$, V vs Ag/$AgNO_3$	ΔE°, mV
0	-0.101	-0.650	549
5	-0.113	-0.642	530
10	-0.109	-0.631	523
20	-0.106	-0.615	509
33	-0.105	-0.596	491
75	-0.102	--	--
100	-0.102	-0.541	439

Cyclic voltammetry of TCNQ in acetonitrile/$BMImBF_4$

%$BMImBF_4$	$E°_1$, V vs Ag/$AgNO_3$	$E°_2$, V vs Ag/$AgNO_3$	ΔE°, mV
0	-0.096	-0.645	549
5	-0.101	-0.635	534
10	-0.103	-0.626	524
20	-0.102	-0.610	508
33	-0.100	-0.593	494
50	-0.101	-0.576	476
75	-0.101	-0.557	456
100	-0.096	-0.523	427

Figure S1. Mixture of acetonitrile with 60% (v/v) $BMImPF_6$. Red: $BMIm^+$, Green: PF_6^-, Blue/white: acetonitrile. Adapted with permission from Acc. Chem. Res. (2007) 40, 1087-1096. Copyright 2007 American Chemical Society.

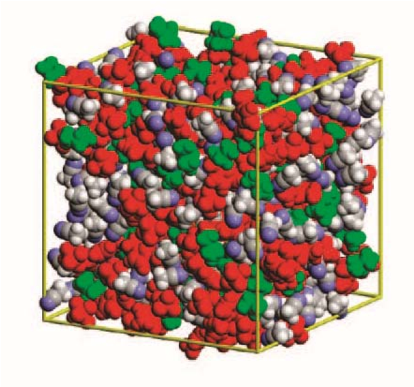

Figure S2. Cyclic voltammetry of TCNQ in $AmNTf_2$, $BMImBF_4$, $BMImPF_6$, $BMImNTf_2$ and acetonitrile. Scan rate: 100 mV/s. Working electrode: Platinum. Acetonitrile contained 0.10 M TBAP

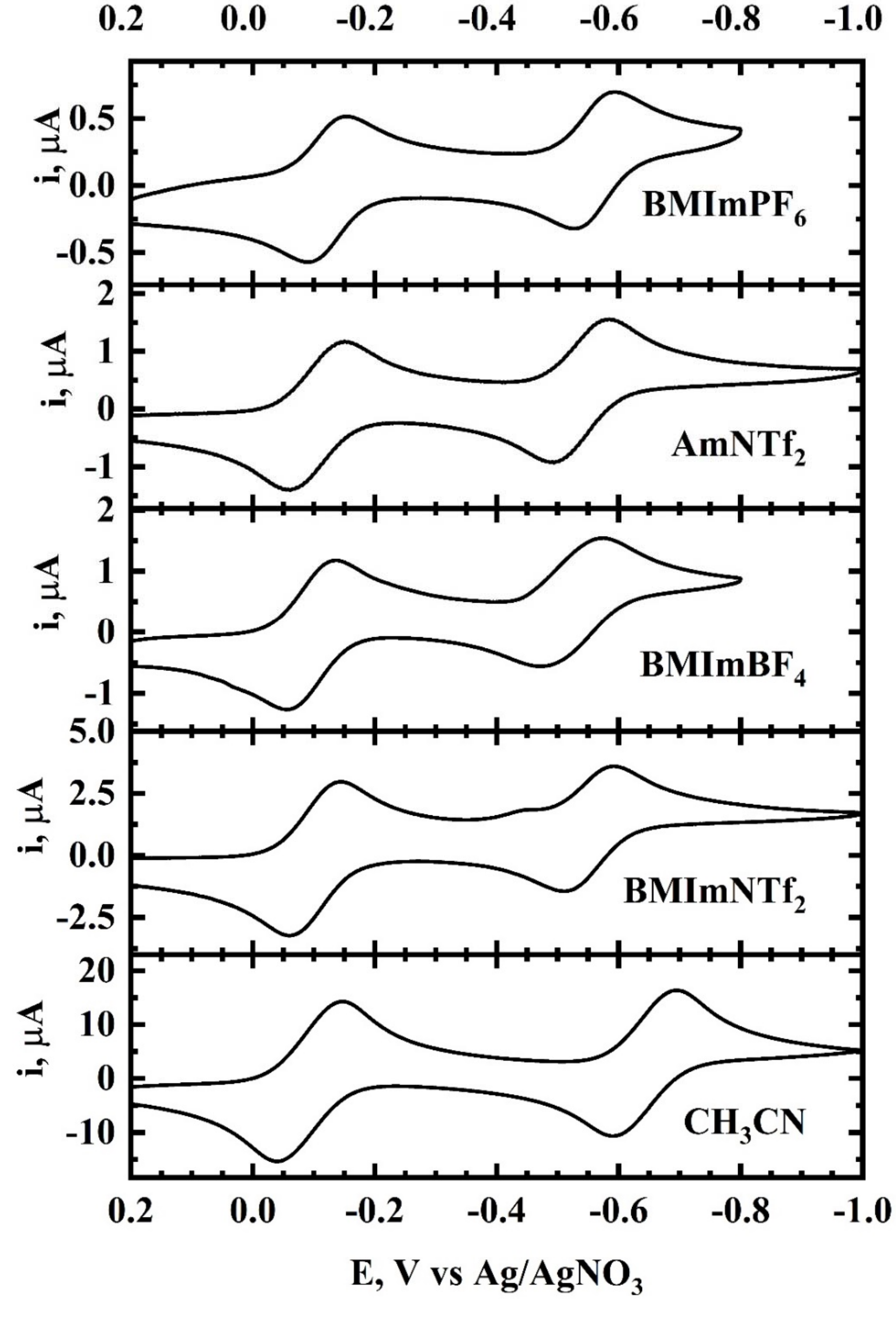

Figure S3. Visible spectroelectrochemistry of TCNQ in acetonitrile. A. First wave. B. Second wave. Electrolyte: 0.10 M TBAP

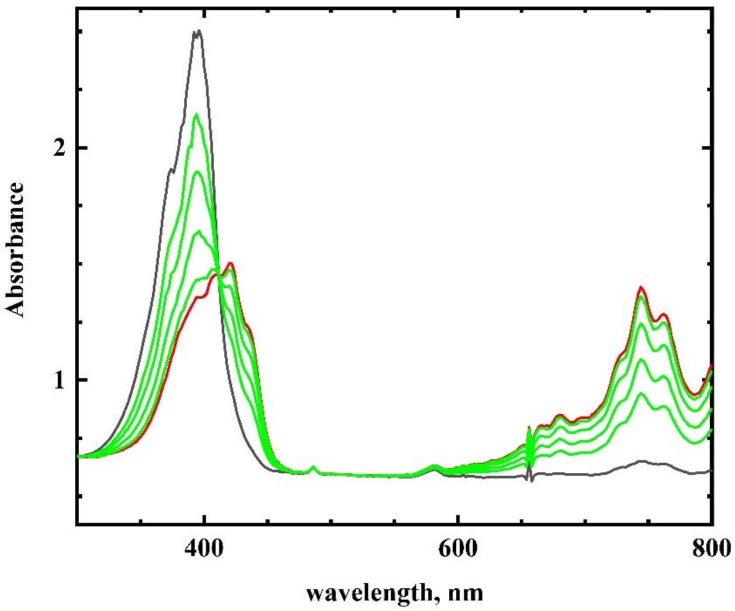

A

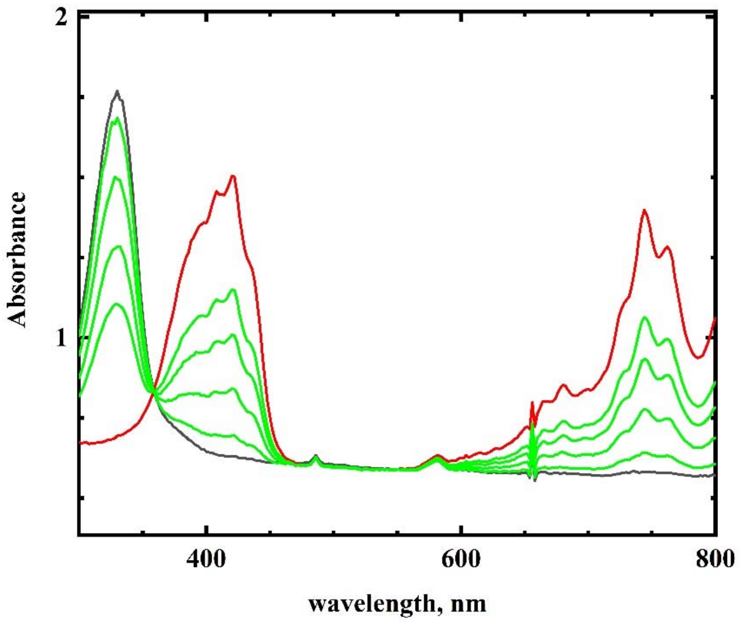

B

Figure S4. Infrared spectroelectrochemistry of TCNQ in THF. Initial difference spectrum (black); $TCNQ^{-\cdot}$ (blue); $TCNQ^{2-}$ (green); end of scan (red). Electrolyte: 0.10 M TBAP

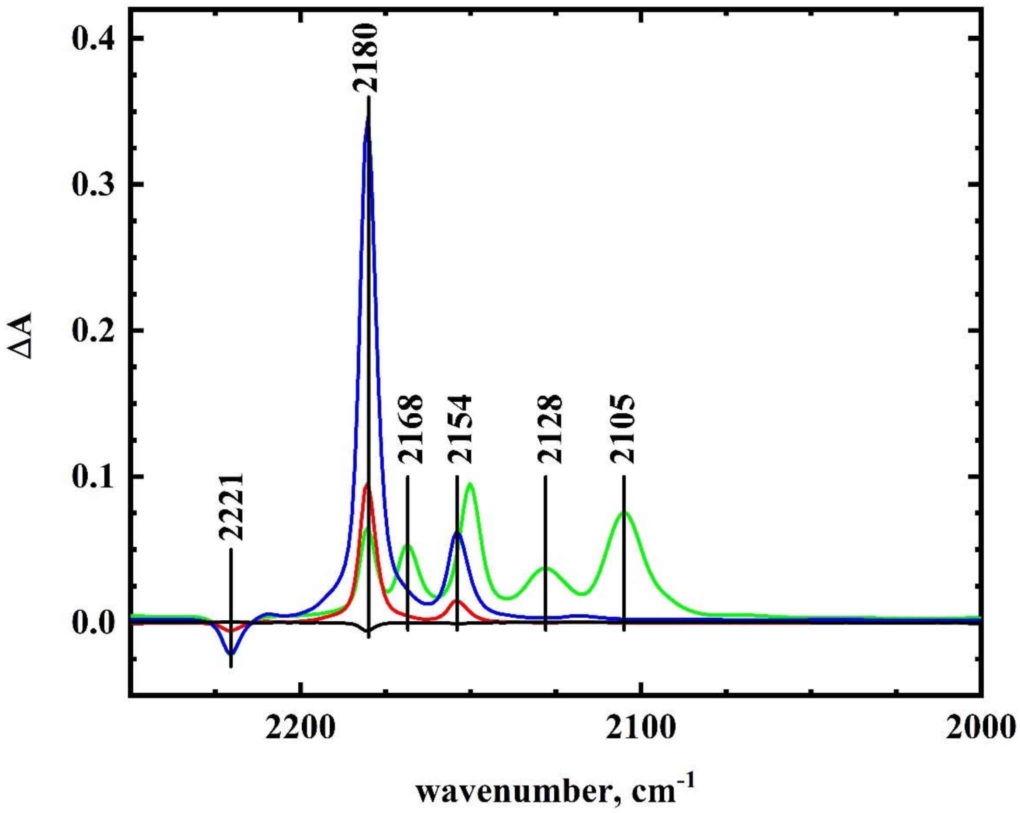

Figure S5. DFT structure of the tetramethylammonium-TCNQ^{2-} ion pair. Gaussian 16.

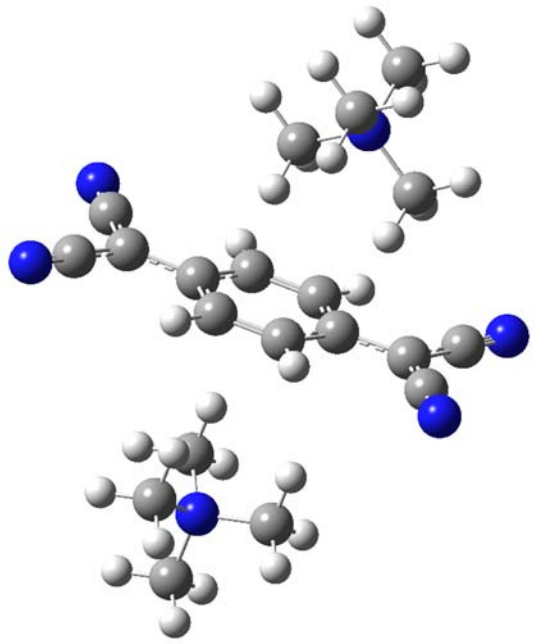

Supplement: Supplementary file 1 [file molecules-25-00303-s001.zip › TCNQ SI revision 2.pdf]
