# Supplementary material for: Voltammetry and Spectroelectrochemistry of TCNQ in Acetonitrile/RTIL Mixtures
Source: Molecules. 2020 Jan 12;25(2):303. doi: 10.3390/molecules25020303 (PMC7024151; doi:10.3390/molecules25020303)
Supplement: Supplementary file 1 [file molecules-25-00303-s001.zip › Rightslink® by Copyright Clearance Center.pdf]

Copyright Clearance Center RightsLink® Home Help Email Support Sign in Create Account

ACS Publications Most Trusted. Most Cited. Most Read.

Molecular Solutes in Ionic Liquids: A Structural Perspective

Author: Agílio A. H. Pádua, Margarida F. Costa Gomes, José N. A. Canongia Lopes
Publication: Accounts of Chemical Research
Publisher: American Chemical Society
Date: Nov 1, 2007

Copyright © 2007, American Chemical Society

PERMISSION/LICENSE IS GRANTED FOR YOUR ORDER AT NO CHARGE

This type of permission/license, instead of the standard Terms & Conditions, is sent to you because no fee is being charged for your order. Please note the following:

- Permission is granted for your request in both print and electronic formats, and translations.
- If figures and/or tables were requested, they may be adapted or used in part.
- Please print this page for your records and send a copy of it to your publisher/graduate school.
- Appropriate credit for the requested material should be given as follows: "Reprinted (adapted) with permission from (COMPLETE REFERENCE CITATION). Copyright (YEAR) American Chemical Society." Insert appropriate information in place of the capitalized words.
- One-time permission is granted only for the use specified in your request. No additional uses are granted (such as derivative works or other editions). For any other uses, please submit a new request.

If credit is given to another source for the material you requested, permission must be obtained from that source.

BACK CLOSE WINDOW

© 2019 Copyright - All Rights Reserved | Copyright Clearance Center, Inc. | Privacy statement | Terms and Conditions
Comments? We would like to hear from you. E-mail us at customercare@copyright.com
